# Introduction to the *BioTM* special issue “Nucleic Acid Delivery: Enabling the Drugs of Tomorrow”

**DOI:** 10.1002/btm2.10039

**Published:** 2016-10-10

**Authors:** Kathryn A. Whitehead

**Affiliations:** ^1^ Depts. of Chemical Engineering and Biomedical Engineering Carnegie Mellon University Pittsburgh PA 15213

It is my pleasure to introduce the second issue of *Bioengineering and Translational Medicine* during its inaugural year. This issue focuses on one of the most pressing questions for drug delivery scientists today: How can we engineer systems that protect the sensitive cargo that is DNA and RNA while simultaneously facilitating intracellular delivery? With continuous advances in our understanding of the biology of nucleic acids and their role in a myriad of diseases, it is becoming increasingly evident that nucleic acid therapeutics will be a crux of the personalized medicines of tomorrow. However, we as a society will not be able to capitalize on their promise until we've developed methods for safe and effective delivery.

This issue highlights nine biomedical and chemical engineering laboratories that have developed unique engineering solutions to the challenges of nucleic acid delivery. Three of these report on new possibilities associated with RNA delivery: Efie Kokkoli and colleagues describe an siRNA‐loaded nanoparticle targeted to the overexpressed α6β4 receptor on cervical cancer cells for selective cancer cell killing.^1^ Danielle Benoit and coworkers explore both the intended and unintended effects of diblock copolymer nanoparticle delivery of siRNA to human mesenchymal stem cells.^2^ Qiaobing Xu and colleagues show that bioreducible lipid nanoparticles can also deliver RNA—this time miRNA—to mesenchymal stem cells.^3^ In their study, they demonstrate the ability of miR‐9 to promote neuronal differentiation following delivery.

The remainder of the issue's articles focus on gene therapy. Jordan Green and members of his lab demonstrate the ability of polymer nanoparticles to induce cancer cell death through the delivery of “TRAIL” (Tumor Necrosis Factor‐Related Apoptosis‐Inducing Ligand) DNA.^4^ Kaushal Rege and his coworkers also report on the ability of TRAIL to kill cancer cells, in this case using aminoglycoside‐derived polymer chemistry for effective DNA delivery.^5^ Dave Lynn and colleagues extend the typical utility of polymer nanoparticle‐mediating DNA delivery to include a controlled release element.^6^

Angela Pannier and coworkers describe the identification of small molecule drugs that enhanced DNA transfection efficacy using a high throughput screening approach.^7^ Stephanie Seidlits, Lonnie Shea, and their colleagues employ gene therapy to reduce neuroinflammation following spinal cord injury.^8^ Specifically, they incorporate a lentiviral delivery system for interleukin‐10 into a multi‐channel bridge that locally delivers DNA to affected tissue following injury. Finally, Morgan Urello, Kristi Kiick, and Millie Sullivan demonstrate the ability of a polymer nanoplex modified with collagen‐mimetic peptides to deliver growth factor‐encoding DNA for accelerated wound healing.^9^

As a whole, this excellent collection of papers advances available technology for RNA and DNA delivery while improving our understanding of intracellular delivery processes. It is my hope that you enjoy reading these contributions as much as I have enjoyed overseeing their publication.

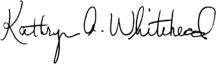

Kathryn A. Whitehead *Depts. of Chemical Engineering and Biomedical Engineering Carnegie Mellon University Pittsburgh, PA, 15213 Email*: kawhite@cmu.edu
